# Neutrophil-to-lymphocyte ratio predicts 30-, 90-, and 180-day readmissions of patients with hepatic encephalopathy

**DOI:** 10.3389/fmed.2023.1185182

**Published:** 2023-06-30

**Authors:** Lin Zhang, Wei Zhang, Jian Wang, Qian Jin, Danli Ma, Rui Huang

**Affiliations:** Peking University People's Hospital, Peking University Hepatology Institute, Beijing Key Laboratory of Hepatitis C and Immunotherapy for Liver Diseases, Beijing International Cooperation Base for Science and Technology on Non-Alcoholic Fatty Liver Disease (NAFLD) Diagnosis, Beijing, China

**Keywords:** hepatic encephalopathy, decompensated cirrhosis, neutrophil-to-lymphocyte ratio, readmission, hospitalization

## Abstract

**Introduction:**

Hepatic encephalopathy (HE) is a significant complication of cirrhosis, known to be associated with hospital readmission. However, few new serological indicators associated with readmission in HE patients have been identified and reported. The objective of our study was to identify simple and effective predictors reated to readmission in HE patients.

**Materials and methods:**

We conducted a retrospective study at a single center on adult patients admitted with HE from January 2018 to December 2022. The primary endpoint was the first liver-related readmission within 30, 90, and 180 days, and we collected electronic medical records from our hospital for sociodemographic, clinical, and hospitalization characteristics. We utilized logistic regression analysis and multiple linear regression analysis to determine the predictors that were associated with the readmission rate and the length of the first hospitalization.

**Results:**

A total of 424 patients were included in the study, among whom 24 (5.7%), 63 (14.8%), and 92 (21.7%) were readmitted within 30, 90, and 180 days, respectively. Logistic regression analysis showed that insurance status, alcoholic liver disease (ALD), ascites, the model for end-stage liver disease (MELD) score, and neutrophil-to-lymphocyte ratio (NLR) were significantly associated with 30-, 90-, and 180-day readmissions. Age and hepatocellular carcinoma (HCC) were predictors of 90- and 180-day readmissions. ALD was identified as a unique predictor of readmission in men, while hypertension was a predictor of 180-day readmission in women. Variceal bleeding, chronic kidney disease, and MELD score were associated with the length of the first hospitalization.

**Conclusions:**

NLR at discharge was identified as a significant predictor of 30-, 90- and 180-day readmissions in patients with HE. Our findings suggest that incorporating NLR into routine clinical assessments could improve the evaluation of the prognosis of liver cirrhosis.

## Introduction

Hepatic encephalopathy (HE) is a significant complication of advanced liver disease that is associated with poor outcomes. The incidence of HE in patients with cirrhosis can reach 11.6/100 person-years (1). Once HE develops, the 1- and 3-year cumulative survival rates are only 50 and 25% (2). HE is characterized by changes in personality, consciousness, cognition, and motor function and is associated with a range of clinical complications (3). Additionally, HE has a negative impact on the health-related quality of life of affected patients (4).

According to reports, the readmission rates of patients with cirrhosis were 20.7 and 30.1% within 30 and 90 days, respectively, with HE being the most common reason for readmission (5). Furthermore, HE was also the leading cause of readmission for patients with decompensated cirrhosis within 180 days (45.4%), with a mortality rate of 35.0% within 180 days (*p* = 0.001) (6). Readmission not only predicts poor outcomes for cirrhotic and HE patients, but it also represents a significant financial burden. A national study conducted in America found that the total healthcare cost for cirrhotic patients with readmission was significantly higher than for those without readmission within 30 days ($64,795 vs. $31,017, *p* < 0.001) (7).

Several factors have been reported as predictors of readmission in HE patients, including insurance status, alcoholic liver disease (ALD), the presence of portal hypertension, international normalized ratio (INR), the model for end-stage liver disease (MELD) score, ascites, receiving paracentesis, and acute kidney injury (1, 7–9). Previous studies reported that volume status, including ascites, receiving paracentesis, and acute kidney injury, were associated with early readmission, as HE patients with refractory ascites and/or recurrent acute kidney injury often require repeated paracentesis (7, 10). Uninsured patients were found to have higher alcohol-related admissions and lower inpatient and 90-day transplant rates, even after controlling for age, gender, race, ethnicity, comorbid conditions, and cirrhosis status (8). INR was found to independently predict early readmission in patients with HE, and MELD score was the only predictor when HE was considered the only cause of readmission (9). Furthermore, portal hypertension was associated with HE, as patients with severe liver disease manifested by portal hypertension had an incidence rate of 27.11 (95% CI, 26.84, 27.38) hospitalizations compared to 4.25 (95% CI, 4.18, 4.31) hospitalizations per person-years for those without, giving an incidence rate ratio (IRR) of 6.38 (95% CI, 6.27, 6.51) (1).

Although the neutrophil-to-lymphocyte ratio (NLR) has been reported to be associated with diseases, such as cardiac and chronic obstructive pulmonary disease (11, 12), as well as hepatopathy-related diseases, such as alcoholic hepatitis, hepatocellular carcinoma (HCC), and others (13–16), its correlation with readmission in HE patients has not been clearly researched. To clarify whether NLR is one of the predictors of readmission in HE patients, we conducted this retrospective study.

## Materials and methods

### Data source

We conducted a retrospective study at a single center, including adult patients admitted with HE between January 2018 and December 2022. The patients were identified through the electronic medical record of our hospital using admission diagnoses of HE with ICD-9 codes 570.X, 572.2, 348.3, 348.31, 348.39, and 291.2. Patients with incomplete information and those who were lost to follow-up were excluded. The study was conducted in accordance with the ethical guidelines of the 1975 Declaration of Helsinki (6th revision, 2008) and was approved by the Ethics Committee of Peking University People's Hospital (No. 2022PHB251-01). Informed consent was obtained from all patients included in the study.

### Baseline characteristics

We collected sociodemographic, clinical, and hospitalization characteristics, including gender, age, insurance (including Chinese medical insurance for residents and Chinese worker medical insurance), baseline liver disease [alcohol or non-alcohol (viral hepatitis, non-alcoholic steatohepatitis, drug-induced liver injury, and autoimmune liver disease)], comorbidities [hypertension, diabetes, and chronic kidney disease (CKD)], and complications [HCC, ascites, variceal bleeding, and spontaneous bacterial peritonitis (SBP)]. Laboratory results at the first discharge, including ammonia, MELD score, and ALD diagnosed as alcohol intake ≥40 g per day in men and ≥20 g per day in women, lasting ≥5 years, were also collected (17). Baseline liver disease (hepatitis B and C, autoimmune liver disease, and drug-induced liver injury), with the exception of alcoholic liver disease, were extracted from admission and discharge diagnoses, and only one occurrence of each diagnosis was recorded. Laboratory data were obtained from the hospital's clinical data center. The MELD score was calculated using total bilirubin, creatinine, INR, and a history of cholestatic liver disease. The NLR was calculated as the ratio of absolute neutrophil count to absolute lymphocyte count.

### Study outcomes

We collected information on readmissions through the electronic medical record of our hospital, which includes medical history, physical examinations, daily notes, laboratory results, and discharge summaries. To capture episodes of hospitalization at other hospitals, we contacted patients who had liver-related readmissions to any hospital since the first hospitalization. Hospitalization characteristics recorded included complications of cirrhosis at the first admission (volume-related complications such as ascites, edema, or SBP; other complications or more than one complication such as HCC, variceal bleeding, and hepatic hydrothorax), cause of readmission (HE, volume-related, other, or more than one complication), and the length of stay for the first hospitalization (calculated from admission to discharge).

We defined the primary endpoint as the first liver-related readmission occurring within 30, 90, and 180 days following the initial hospitalization. Liver-related readmission was defined as readmission due to any of the following: HE, volume-related complications (ascites, edema, and SBP), HCC, variceal bleeding, and hepatic hydrothorax. HE was diagnosed according to the West Haven criteria and patients' clinical manifestations and ammonia levels. Ascites were diagnosed by imaging examination or clinical percussion revealing fluid in the peritoneal cavity. HCC was diagnosed based on pathology or imaging examination. Variceal bleeding was diagnosed based on clinical features and endoscopy. SBP was diagnosed when the ascitic fluid neutrophil count exceeded 250/mm^3^. Edema was diagnosed based on patient complaints and pitting edema of both lower limbs. Hepatic hydrothorax was diagnosed based on an imaging examination. Patients could have more than one liver-related reason for admission, and all other readmissions were excluded from the analysis.

### Statistical analysis

Continuous variables were presented as mean ± standard deviations (SD) and categorical variables as count with percentage. Differences between groups were analyzed using Student's *t*-test for age, length of stay, MELD, NLR, and ammonia at discharge, and chi-square test for insurance, baseline liver disease, comorbidities, complications, treatment, and cause of readmission. Predictors associated with readmission and the length of the first hospitalization were analyzed using logistic regression analysis and multiple linear regression analysis, respectively. Statistical significance was considered at *p* < 0.05. Data analyses were performed using SPSS version 23.0 (IBM Corp, Armonk, NY, USA).

## Results

### Baseline sociodemographic and clinical characteristics

We included 424 patients admitted with HE, and their baseline characteristics are presented in Table 1. Within 30, 90, and 180 days, 24 (5.7%), 63 (14.8%), and 92 (21.7%) patients were readmitted, respectively. Among the patients, 283 (66.7%) were male patients, and the mean age was 59.9 ± 11.5 years; 120 (28.3%) patients had alcoholic cirrhosis. There were 280 patients classified as grade 0, 66 were of grade 1, 42 were of grade 2, 33 were of grade 3, and 1 was of grade 4. At baseline, 40 (9.4%), 246 (58.0%), 67 (15.8%), and 20 (4.7%) patients had HCC, ascites, variceal bleeding, and SBP, respectively. Patients who were readmitted within 180 days were older (62.0 ± 9.6 vs. 59.3 ± 12.0, *p* = 0.025) and had a higher proportion of no insurance [25 (27.2%) vs. 52 (15.7%), *p* = 0.010], ALD [34 (37.0%) vs. 86 (25.9%), *p* = 0.027], CKD [21 (22.8%) vs. 39 (11.7%), *p* = 0.007], HCC [15 (16.3%) vs. 25 (7.5%), *p* = 0.012], and ascites [65 (70.7%) vs. 181 (54.5%), *p* = 0.004] than those who were not readmitted within 180 days.

**Table 1:** Baseline characteristics of included patients.

**Characteristics**	**All (*n* = 424)**	**30-day readmission (*n* = 24)**	**90-day readmission (*n* = 63)**	**180-day readmission (*n* = 92)**
**Age (years), mean** **±SD**	59.9 ± 11.5	62.8 ± 11.0	61.9 ± 9.9	62.0 ± 9.6^*^
**Male**, ***n*** **(%)**	283 (66.7)	15 (62.5)	43 (68.3)	63 (68.5)
**Insurance**, ***n*** **(%)**				
Any insurance	347 (81.8)	17 (70.8)	47 (74.6)	67 (72.8)
No Insurance	77 (18.2)	7 (29.2)	16 (25.4)	25 (27.2)^*^
**Baseline liver disease**, ***n*** **(%)**				
Alcohol	120 (28.3)	10 (41.7)	23 (36.5)	34 (37.0)^*^
Non-alcohol	304 (71.7)	14 (58.3)	40 (63.5)	58 (63.0)
**Comorbidities**, ***n*** **(%)**				
Hypertension	130 (30.6)	11 (45.8)	21 (33.3)	28 (30.4)
Diabetes	123 (29.0)	6 (25.0)	22 (34.9)	33 (35.9)
Chronic kidney disease	60 (14.2)	8 (33.3)^*^	16 (25.4)^*^	21 (22.8)^*^
**Complications**, ***n*** **(%)**				
Hepatocellular carcinoma	40 (9.4)	3 (12.5)	12 (19.0)^*^	15 (16.3)^*^
Ascites	246 (58.0)	20 (83.3)^*^	45 (71.4)^*^	65 (70.7)^*^
Variceal bleeding	67 (15.8)	5(20.8)	16 (25.4)^*^	20 (21.7)
Spontaneous bacterial peritonitis	20 (4.7)	2 (8.3)	2 (3.2)	4 (4.3)
**Treatment**, ***n*** **(%)**				
Lactulose alone	250 (58.9)	13 (54.2)	40 (63.5)	57 (62.0)
Lactulose + rifaximin	17 (4.0)	1 (4.2)	3 (4.8)	3 (3.3)
Rifaximin alone	81 (19.1)	9 (37.5)	17 (27.0)	22 (23.9)
Other	76 (17.9)	1 (4.2)	3 (4.8)	10 (10.9)

Non-alcoholic liver disease: viral hepatitis, non-alcoholic steatohepatitis, drug-induced liver injury, or autoimmune liver disease.

^*^Comparisons between patients readmitted with and without 30, 90, and 180 days, p < 0.05.

### Hospitalization characteristics

Patients were admitted for liver-related reasons other than HE, including volume-related (247, 58.3) and other complications (177, 41.7%). The mean length of hospitalization for all patients at the time of admission was 17.9 ± 12.1 days. HE was the most common reason for readmission, with 15 (62.5%), 30 (47.6%), and 48 (52.1%) patients being readmitted for HE within 30, 90, and 180 days, respectively. Furthermore, patients who were readmitted had a higher NLR level at their first discharge (8.17 ± 12.1 vs. 3.95 ± 4.96; 7.17 ± 9.72 vs. 3.67 ± 4.44; 6.80 ± 8.62 vs. 3.47 ± 4.28, *p* < 0.001) compared to those without readmission in 30, 90, or 180 days (see Table 2). Patients who were readmitted within 30 days had the longest length of stay during their first hospitalization, which was significantly higher than those without readmission in 30 days (24.8 ± 19.2 vs. 17.7 ± 11.90, *p* < 0.001).

**Table 2:** Hospitalization characteristics.

**Index hospitalization**	**All**	**30-day readmission**		**90-day readmission**				**180-day readmission**		
		**Not readmitted**	**Readmitted**	***p*** **-value**	**Not readmitted**	**Readmitted**	***p*** **-value**	**Not readmitted**	**Readmitted**	***p*** **-value**
	***n*** = **424**	***n*** = **400**	***n*** = **24**		***n*** = **361**	***n*** = **63**		***n*** = **332**	***n*** = **92**	
**Other complications of cirrhosis at the first admission**, ***n*** **(%)**										
Volume-related	247 (58.3)	227 (56.8)	20 (83.3)	0.010	202 (56.0)	45 (71.4)	0.026	182 (54.8)	65 (70.7)	0.008
Other or > 1 complication	177 (41.7)	173 (43.2)	4 (16.7)		159 (44.0)	18 (28.6)		150 (45.2)	27 (29.3)	
**The length of stay (days), mean** **±SD**	17.9 ± 12.1	17.5 ± 11.4	24.8 ± 19.2	0.004	17.7 ± 11.8	19.1 ± 13.6	0.389	17.6 ± 11.9	18.9 ± 12.6	0.347
**MELD score at discharge, mean** **±SD**	13.5 ± 8.9	13.0 ± 8.8	21.4 ± 6.3	< 0.001	12.6 ± 8.8	19.0 ± 7.2	< 0.001	12.4 ± 8.9	17.7 ± 7.4	0.060
**NLR at discharge, mean** **±SD**	4.19 ± 5.68	3.95 ± 4.96	8.17 ± 12.1	< 0.001	3.67 ± 4.44	7.17 ± 9.72	< 0.001	3.47 ± 4.28	6.80 ± 8.62	< 0.001
**Ammonia at discharge (μmol/L), mean** **±SD**	71.2 ± 33.7	71.2 ± 34.0	71.8 ± 30.7	0.934	71.8 ± 34.2	68.1 ± 30.5	0.429	70.6 ± 31.3	73.5 ± 41.2	0.466
**The cause of readmission**, ***n*** **(%)**										
Hepatic encephalopathy		—	15 (62.5)		—	30 (47.6)		—	48 (52.1)	
Volume-related		—	5 (20.8)		—	22 (34.9)		—	20 (21.7)	
Other or > 1 complication		—	4 (16.7)		—	11 (17.4)		—	24 (26.1)	

Volume-related: ascites, edema, or spontaneous peritonitis. Other or >1 complication: hepatocellular carcinoma, variceal bleeding, hepatic hydrothorax, acute kidney injury, and electrolyte abnormalities or more than one complication.

MELD, model for end-stage liver disease; NLR, neutrophil-to-lymphocyte ratio.

### Predictors of 30-, 90-, and 180-day readmissions

Table 3 displays the results of logistic regression analysis for predictors of readmission within 30, 90, and 180 days. Significant predictors of readmission included no insurance, ALD, ascites, MELD score, and NLR at first discharge for all three time periods. Age and HCC were also significant predictors of readmission at 90 and 180 days.

**Table 3:** Predictors of 30-, 90-, and 180-day readmissions.

**Characteristics**	**β**	***p*-value**	**OR**	**95% CI**
**30-day readmission**				
No insurance	1.155	0.047	3.173	1.015, 9.915
Alcoholic liver disease	1.302	0.037	3.675	1.081, 12.491
Ascites	2.019	0.006	7.529	1.780, 31.855
MELD score at discharge	0.191	< 0.001	1.210	1.125, 1.302
NLR at discharge	0.058	0.044	1.060	1.002, 1.122
**90-day readmission**				
Age	0.036	0.024	1.036	1.005, 1.069
No insurance	0.770	0.048	2.159	1.006, 4.633
Alcoholic liver disease	0.821	0.035	2.274	1.061, 4.873
Hepatocellular carcinoma	1.044	0.022	2.841	1.162, 6.945
Ascites	0.772	0.036	2.164	1.051, 4.456
MELD score at discharge	0.165	< 0.001	1.180	1.122, 1.240
NLR at discharge	0.066	0.008	1.068	1.017, 1.112
**180-day readmission**				
Age	0.040	0.003	1.041	1.013, 1.069
No insurance	1.003	0.003	2.727	1.415, 5.256
Alcoholic liver disease	0.932	0.006	2.539	1.311, 4.915
Hepatocellular carcinoma	0.891	0.031	2.438	1.085, 5.476
Ascites	0.701	0.022	2.016	1.105, 3.679
MELD score at discharge	0.141	< 0.001	1.151	1.102, 1.203
NLR at discharge	0.077	0.002	1.080	1.030, 1.133

MELD, model for end-stage liver disease; NLR, neutrophil-to-lymphocyte ratio.

However, gender differences were observed in the predictors of readmission. In men (Table 4), in addition to HCC, ascites, MELD score, and NLR at discharge, ALD was a significant predictor of 30-, 90-, and 180-day readmissions. Interestingly, ALD was not a significant predictor of readmission in women, while hypertension was significantly associated with 180-day readmission in women (Table 5).

**Table 4:** Predictors of 30-, 90-, and 180-day readmissions in men.

**Characteristics**	**β**	***P***	**OR**	**95% CI**
**30-day readmission**				
Alcoholic liver disease	1.423	0.043	4.150	1.043, 16.521
Ascites	2.710	0.027	15.031	1.372, 164.729
MELD score at discharge	0.218	< 0.001	1.244	1.128, 1.372
**90-day readmission**				
Alcoholic liver disease	0.915	0.028	2.496	1.106, 5.635
Hepatocellular carcinoma	1.171	0.028	3.224	1.134, 9.170
MELD score at discharge	0.184	< 0.001	1.202	1.128, 1.280
**180-day readmission**				
Alcoholic liver disease	0.966	0.008	2.627	1.291, 5.348
Ascites	1.292	0.003	3.640	1.561, 8.466
MELD score at discharge	0.162	< 0.001	1.176	1.112, 1.244
NLR at discharge	0.072	0.022	1.075	1.010, 1.144

MELD, model for end-stage liver disease; NLR, neutrophil-to-lymphocyte ratio.

**Table 5:** Predictors of 30-, 90-, and 180-day readmissions in women.

**Characteristics**	**β**	***P***	**OR**	**95% CI**
**30-day readmission**				
No insurance	3.006	0.013	20.209	1.861, 219.444
MELD score at discharge	0.264	0.005	1.302	1.082, 1.567
**90-day readmission**				
Age	0.075	0.023	1.077	1.010, 1.149
MELD score at discharge	0.135	0.006	1.144	1.039, 1.260
**180-day readmission**				
Age	0.085	0.002	1.089	1.031, 1.149
No insurance	1.745	0.007	5.725	1.609, 20.376
Hypertension	1.500	0.017	4.481	1.309, 15.331
MELD score at discharge	0.124	0.007	1.131	1.035, 1.237
NLR at discharge	0.109	0.039	1.115	1.006, 1.237

MELD, model for end-stage liver disease; NLR, neutrophil-to-lymphocyte ratio.

### Predictors of the length of hospitalization at the first admission

Table 6 shows that variceal bleeding (*p* = 0.006), CKD (*p* = 0.003), and MELD score at discharge (*p* = 0.024) were significant predictors of the length of hospitalization for patients at their first admission.

**Table 6:** Predictors of the length of hospitalization at the first admission.

**Characteristics**	**B**	**SE**	**β estimate**	***p***	**95.0% CI for β**
Constant	16.046	3.917		0.000	8.346, 23.746
Age	−0.065	0.053	−0.063	0.219	−0.169, 0.039
Men	0.460	1.319	0.018	0.727	−2.133, 3.052
No insurance	−1.838	1.465	−0.060	0.210	−4.717, 1.042
Alcoholic liver disease	−2.577	1.374	−0.099	0.061	−5.279, 0.124
Hepatocellular carcinoma	0.467	1.919	0.012	0.808	−3.305, 4.239
Ascites	1.741	1.168	0.073	0.137	−0.555 ± 4.038
Spontaneous bacterial peritonitis	4.264	2.765	0.076	0.124	−1.172, 9.699
Variceal bleeding	4.402	1.593	0.135	0.006	1.269, 7.534
Chronic kidney disease	4.973	1.658	0.149	0.003	1.713, 8.232
Diabetes	−0.422	1.242	−0.016	0.734	−2.864, 2.019
Hypertension	1.789	1.272	0.070	0.160	−0.711, 4.289
Ammonia at discharge	0.020	0.017	0.056	0.249	−0.014, 0.053
MELD score at discharge	0.204	0.090	0.111	0.024	0.026, 0.381
N/L at discharge	0.064	0.102	0.031	0.530	−0.136, 0.264

MELD, model for end-stage liver disease; NLR, neutrophil-to-lymphocyte ratio; NLR, neutrophil-to-lymphocyte ratio.

## Discussion

Patients who are readmitted for HE are at an increased risk of morbidity and mortality, making readmissions costly for both patients and healthcare systems. Our study found that NLR at discharge was a significant predictor of 30-, 90-, and 180-day readmissions for patients with HE. This new predictor offers valuable insights that can inform clinical decision-making and improve the evaluation of the prognosis of liver cirrhosis. Additionally, our study showed that CKD, one of the most important comorbidities, was a significant factor affecting the length of hospitalization at the first admission for patients with HE. Therefore, managing comorbidities in addition to treating HE can help reduce the length of hospital stay for these patients.

We found that NLR at the first discharge was a crucial predictor of 30-, 90-, and 180-day readmissions. As neutrophils play an important role in many types of liver disease and inappropriate activation and homing of neutrophils to the microvasculature can induce immune-mediated liver injury, NLR is a serological marker that reflects the level of inflammation and is superior to simple white blood cell counts in cirrhotic patients (18). It was reported that NLR could correctly identify these patients who had a high risk of mortality despite low MELD scores and a high risk of mortality (11), and NLR was positively correlated with Child–Turcotte–Pugh (CTP) score. In patients with Child–Pugh class C, NLR is an independent predictor of poor 1-month survival (13). A high NLR reflects the severity and progression of cirrhosis and has been associated with an increased risk of mortality in patients with alcoholic hepatitis and HE (14, 15). It has been reported that for every 1-unit increase in NLR at admission, the increased risk of death in patients with alcoholic hepatitis was 20 and 9% in 30 days and 12 months, respectively (16). Another study reported that elevated NLR was significantly associated with an increased risk of 30-day mortality in HE patients (14). As NLR reflects the level of inflammation, it may aggravate the symptoms of HE by enhancing ammonia-induced neurotoxicity through the blood–brain barrier (19). Elevated plasma levels of inflammatory markers in patients with HE are correlated with the severity of HE and are not determined by the severity of underlying liver disease or ammonia levels (20). Additionally, the neutrophil count is higher in decompensated cirrhotic patients than in compensated cirrhotic patients, while the lymphocyte count is lower in decompensated cirrhosis (21). This suggests that a higher level of NLR reflects worse patient status in decompensated cirrhosis. In patients with HCC, NLR has been identified as an independent factor for worse survival and predicts the response to treatment (18). Low NLR was significantly associated with a better survival rate and recurrence-free or disease-free survival (22). A review of the association between NLR and SBP concluded that NLR was a valid biomarker that can be readily integrated into clinical settings to prevent and predict SBP in cirrhotic patients (23). NLR has also been found to be a predictor of survival and readmission in other diseases such as cardiac and chronic obstructive pulmonary disease (11, 12). To the best of our knowledge, our study is the first to report the predictive role of NLR in the readmission of patients with HE. The correlation between NLR and readmission suggests that controlling inflammation in HE patients in clinical practice can help reduce the liver disease-related readmission rate in HE patients.

In our study, demographic variables, except for age and insurance, were not found to be significant predictors of readmission. It is worth noting that in China, not all individuals have access to medical insurance. Lack of medical insurance can hinder access to timely treatment and routine examinations, which may lead to a worsened condition and an increased risk of readmission. This is consistent with previous research by Andrew, who identified age and medical insurance as social-demographic predictors of 30-day readmission in patients with HE (7). Our study further found that insurance status was a significant predictor of readmission within 180 days. Therefore, access to medical insurance is crucial for patients with HE to receive timely and appropriate medical care and reduce the risk of readmission.

Regarding patients' complications and comorbidities, our study found that HCC and ascites were significantly related to HE patients' readmission. Ascites is a common complication of decompensated cirrhosis, and it directly increases the risk of further complications such as SBP, umbilical hernias, and respiratory compromise, all of which may require readmission (24). A previous study concluded that ascites were associated with early readmission in patients with cirrhosis (25). Ascites production is related to portal hypertension and splanchnic vasodilation, and it can also lead to renal dysfunction and hepatorenal syndrome (26). Previous research has suggested that ascites and renal dysfunction played important roles in the outcomes of patients with cirrhosis, and the use of diuretics and renal failure may impact HE as well (19, 26). Meanwhile, HCC is difficult to treat and associated with high mortality rates and surgical morbidity, increasing the risk of readmission (27). Hepatic resection and transplantation of HCC patients are still associated with a high risk of mortality and postoperative readmission rates. Furthermore, these procedures subject patients to higher medical and surgical morbidities compared to those encountered in the general surgery population (28). These complications and comorbidities can undoubtedly aggravate the condition of HE patients. In view of gender differences, ALD was a significant predictor of readmission in men. ALD has been found to be an important predictor of readmission in patients with cirrhosis and HE (29), and alcohol abuse is a common reason for readmission in cirrhotic patients (30). A study reported that in patients with alcoholic liver cirrhosis, there was a steady rise in the risk-adjusted 30-day all-cause readmission rate as well as alcoholic liver cirrhosis-specific readmission rate and readmission proportion (31). Furthermore, the increasing rate of readmission in ALD patients also increases the total hospital cost and the total days of hospital stay. Effective alcohol use disorder interventions can help reduce costs related to inpatient cirrhosis management (32). Managing HCC and ALD patients well in clinical practice can reduce the risk of readmission for patients with HE.

MELD score was used to identify prognosis and survival in patients with cirrhosis (33). Previous studies found that MELD score, INR, and hemoglobin were predictors of early readmission in cirrhotic patients after the resolution of HE (9). We did not include INR as a single factor, as the MELD score at discharge could represent the HE patients' status.

In our study, we observed gender differences in the predictors of readmission for patients with HE. In addition to ascites, MELD score, and NLR at the first discharge, ALD was significantly associated with readmission in men. Interestingly, hypertension, instead of ALD, predicted long-term readmission in women. Gender differences have been observed in many studies on chronic liver disease and HE. For example, women may experience a more favorable clinical course than men in early chronic liver disease, with sex hormones believed to have a protective effect on fibrosis progression (34, 35). Women with HE exhibited better cognitive performance than men. In a study on gut microbial composition in HE patients, it was observed that certain pathways and microbiota, such as Lactobacillaceae, androstenedione degradation, and cell wall synthesis, remained different between the sexes. These differences may contribute to the disparity in cognition between men and women with cirrhosis and ultimately affect the morbidity of HE in each gender (36). It should be noted that only 141 (33.3%) participants were women, and 9, 20, and 29 female patients were readmitted in 30, 90, and 180 days, so the smaller proportion of female patients may also be a contributing factor to the observed results. Therefore, large-scale studies are needed to further confirm the gender differences in the readmission of HE that we have observed.

Our study has identified NLR as a novel predictor of readmission in HE patients, improving the diagnosis of HE. The management of comorbidities, such as hypertension and CKD, is also crucial to the healthcare of patients with HE. However, our study has some limitations. First, although we took measures to ensure the accuracy of our data by conducting follow-ups with patients, as a retrospective single-center study, to confirm our results, multicenter studies should be carried out in the future. Moreover, we did not explore the mechanisms underlying the relationship between NLR and readmission or potential interventions to reduce readmission rates. Additionally, our study did not include other treatments, such as anti-infection and diuresis, which may become confounding factors for HE readmission. Anti-infection treatment may also affect NLR, and the lack of data on other inflammation indicators, such as CRP and IL-6, is another limitation. Furthermore, we did not collect information on HE patients' comorbidities and complications, such as dehydration, diuretic usage, human albumin infusion, and malnutrition, which may have influenced our findings. Therefore, future studies should aim to address these limitations and better understand these aspects.

## Data availability statement

The original contributions presented in the study are included in the article/supplementary material, further inquiries can be directed to the corresponding author.

## Ethics statement

The studies involving human participants were reviewed and approved by the Ethics Committee of Peking University People's Hospital. Written informed consent for participation was not required for this study in accordance with the national legislation and the institutional requirements.

## Author contributions

RH designed the work and reviewed the article. LZ and WZ interpreted the data and drafted the manuscript. LZ, WZ, JW, QJ, and DM collected the data. All authors read and approved the final manuscript.
